# Anti-müllerian Hormone During Natural Cycle Presents Significant Intra and Intercycle Variations When Measured With Fully Automated Assay

**DOI:** 10.3389/fendo.2018.00686

**Published:** 2018-11-27

**Authors:** Laura Melado, Barbara Lawrenz, Junard Sibal, Emmanuel Abu, Carol Coughlan, Alfredo T. Navarro, Human Mousavi Fatemi

**Affiliations:** ^1^IVF department, IVIRMA Middle-East Fertility Clinic, Abu Dhabi, United Arab Emirates; ^2^Women's University Hospital Tuebingen, Tuebingen, Germany; ^3^IVI Foundation, IVIRMA Valencia, Valencia, Spain

**Keywords:** Anti-Müllerian hormone (AMH), natural cycle, intra-cycle variations, inter-cycle variations, fully automated assay

## Abstract

Anti-Müllerian hormone (AMH) is an important ovarian reserve marker for baseline assessment and therapeutic strategy in fertility treatments, which is considered reliable when measured on any day of the cycle. Recent data have pointed toward significant fluctuations of AMH and questioned whether a single measurement is reliable for clinical decision-making. The aim of this study was to evaluate whether the AMH does have significant variations during a natural cycle when a fully automated assay is used for the sample analysis. We performed a prospective study including healthy volunteers with regular cycles, from April to December 2017. Blood samples for AMH, FSH, LH, estradiol, and progesterone were obtained on day 2/3, day 10, day of LH surge, luteal phase and day 2/3 of subsequent menses. AMH analysis was performed with Elecsys® AMH automated assay. Trial was registered with clinical.trials.gov: NCT03106272. One hundred samples from 22 women with a mean age of 30.74 ± 0.11 years and a BMI of 23.23 ± 0.63 kg/m2 were analyzed. There was a substantial longitudinal fluctuation in AMH levels, indicated by the coefficient of variation (CV) intra-cycle of 0.2070 ± 0.143. A positive correlation between LH and AMH concentrations was found at the moment of LH rise (*p* < 0.0001). Absolute intra-individual inter-cyclic variability was 0.75 ng/mL (range: 0.03–2.81 ng/mL) and inter-cycle CV was 0.28 (Confidence interval: 0.16–0.39; *p* < 0.0001). According to our results, with the use of a fully automated assay in natural cycle, AMH shows significant intra- and inter-cycle variations, which are not caused by analytical variability. Future investigations, evaluating AMH dynamics and the best time for AMH assessment should be conducted.

## Introduction

Anti-Müllerian hormone (AMH) is a dimeric glycoprotein produced by granulosa cells of pre-antral and small antral follicles in the ovary and its production is independent of follicle stimulating hormone (FSH) (1). The release of AMH from the granulosa cells results in measurable serum levels and these concentrations have been shown to be proportional to the number of developing follicles in the ovaries (2). Therefore, AMH has emerged as one of the most important clinical indirect markers for ovarian reserve (3) and is used by clinicians for predicting ovarian response to hyperstimulation for IVF, facilitating counseling of patients and individualization of stimulation regimens. Furthermore, a recent publication demonstrated a strong positive, age-independent relationship between AMH level and number of euploid blastocysts obtained following IVF/ICSI cycles (4), also with miscarriage rates (5).

It is well recognized that serum AMH displays a high inter-individual variability mainly attributed to the different number in antral follicles within groups of women of similar age (2). It also appears to vary with factors such as hormonal contraceptive use, pregnancy, body mass index, smoking (6), and the use of gonadotropins for ovarian stimulation (7, 8). Serum AMH is generally believed to be a reliable test that may be measured on any day of a natural cycle, with minimal intra-individual variability (6, 9–13). However, recent publications have raised doubts as to the reliability of a single AMH test taken *ad-hoc* in a natural cycle. Gorkem et al. demonstrated that serum AMH levels seem to be higher during the follicular phase as compared to the luteal phase in infertile women with normal, high, and low ovarian reserve (14). Other authors have described important variability in AMH concentrations during the menstrual cycle, which was deemed higher than the fluctuations expected due to the analysis alone (15, 16). Moreover, it has been proposed that young women may have a pattern of intra-cycle fluctuation that differs from that of older women (17). Circadian fluctuations for AMH serum levels have also been identified (18).

These fluctuations in AMH during the natural cycle were previously attributed to analytical variations caused by different conditions used for sample storage and/or the assay method (19). But now, AMH can be analyzed with fully automated AMH assays which are highly sensitive and precise, with a broad linear range (20) allowing for more efficient sample processing and reducing possible procedural errors (20–22).

Nowadays, it is widespread accepted that a single AMH measurement as an accurate reflection of a patients' ovarian reserve and clinicians take this result into serious consideration when counseling women about their reproductive health. In light of recent publications demonstrating AMH variability both throughout the menstrual cycle and between consecutive cycles, questions rise as to whether a single AMH measurement would be reliable. The aim of this study was to investigate AMH serum levels in healthy, cycling women using fully automated assay Elecsys^Ⓡ^ AMH (Roche, for Cobas 601 platform^Ⓡ^), at defined time-points of the natural cycle and short-term inter-cycle variations.

## Materials and methods

### Subjects

Twenty-two healthy volunteers were included in this study, which was undertaken from April to December 2017, in IVIRMA Abu Dhabi clinic. Women between the ages of ≥18 to ≤ 38 years old, with regular menstrual cycles between 28 and 32 days and BMI between ≥18 and ≤ 28 kg/m2 were included. Exclusion criteria included the following factors: the intake of hormonal contraceptives for a minimum of 2 months immediately prior to study commencement, pregnancy, breastfeeding, and previous conditions which may adversely affect ovarian reserve (ovarian surgery, chemotherapy, pelvic radiation). No volunteers with infertility background were included. The participants fulfilled a written questionnaire including menstrual cycle pattern, previous pregnancies, deliveries and miscarriages, ethnicity, smoking and possible exclusion criteria. They were informed about the methods, sample processing and objectives of the study and all of them signed informed consent.

### Blood sampling

Blood samples were obtained by venepuncture in the clinic between 8 am and 2 pm, transferring the samples immediately to the laboratory located in the same center for immediate processing and further analysis. During the natural cycle, samples were collected on day 2/3 (AMH_01), day 10 (mid follicular phase, AMH_MFP), day of LH rise (AMH_LHR), mid luteal phase (AMH_MLP), and day 2/3 of the subsequent menstruation (AMH_02). The LH surge was defined for the purpose of our study to have begun when the concentration of LH rose by 180% above the latest serum value available in that patient and continued to rise thereafter (23). For this purpose, LH, estradiol and progesterone were monitored starting on day 10 of a natural cycle and then every 3 days until LH surge was diagnosed (24, 25). The luteal phase was confirmed by an elevated serum progesterone level (>3 ng/ml) 8 days after LH surge.

One sample of 5 mL of blood was collected each time. Blood was centrifuged and serum separated in two parts: one for AMH analysis and one for gonadotropin and steroid hormone analysis. Analysis of FSH, LH, estradiol and progesterone was performed using a competitive immunoassay manufactured by Roche on the Cobas 601 platform®. Results for these hormones were obtained and evaluated on the same day of blood collection. Serum samples for AMH were aliquoted, frozen at −20°C the same day of collection and stored for batch analysis. All samples from each study participant were analyzed under the same conditions on the same day, using Elecsys® AMH automated assay (for Cobas 601 platform, Roche®) according to manufacturer‘s instructions. Imprecision expected from the assay was <5%, as described by the manufacturer; intra-assay and inter-assay co-efficient of variation for Elecsys® AMH automated assay has been reported as 0.5–1.4 and 0.7–1.9%, respectively (26).

### Statistical analysis

A research database was generated for all the variables that were evaluated. The export data were correctly anonymized and analyzed with SPSS 22.0.

Demographic variables and hormonal values were summarized with mean and standard deviation. In order to describe hormones and their dynamics, we determined the mean value and standard deviations each time and represented it with boxplot figures. Moreover, we performed polynomial interpolation for dynamic values and represented the variations with mean polynomial interpolation and variance band.

AMH values were represented for AMH dynamic description using a 4-degree-polynomial-exponential interpolation of data. This polynomial-exponential interpolation was used as well to impute missing data (10 missing data points). Patients were not categorized according to initial AMH since homogeneous variability in cluster groups could not be previously assumed.

Pearson correlation test was performed between AMH values and other hormones along with temporary marks and clustering analyses were done for correlation between AMH dynamic patterns and demographics parameters. For the evaluation of any AMH dynamic patterns related with demographics parameters, we applied clustering analyses over AMH polynomial coefficients that were previously calculated. A *p*-value of 0.05 was considered statistically significant.

### Ethical approval

The study was approved by the internal Ethics Committee under the number REFA011 and registered for clinical.trials.gov: NCT03106272.

## Results

A total of 100 samples from the 22 women were analyzed, with a mean age of 30.7 ± 4.11 years and a BMI of 23.2 ± 3.63 Kg/m^2^ (Table 1). All the samples were analyzed with Elecsys® AMH automated assay for Cobas 601 platform (Roche®).

**Table 1:** Baseline characteristics of participants.

**AGE (Years)**	
Mean (SD)	30.7 ± 4.11
Median (IQR)	30 (22-38)
**BMI (Kg/m**^2^**)**	
Mean (SD)	23.2 ± 3.63
Median (IQR)	23.1 (18.7–27.9)
**RACE, n (%)**	
Asian	11 (50)
Caucasian	4 (18.2)
Arabic	7 (31.8)
**SMOKING, n (%)**	
Non smokers	21 (95.5)
Smokers	1 (4.5)

### Intra-cycle and inter-cycle variations

**Figure 1:**
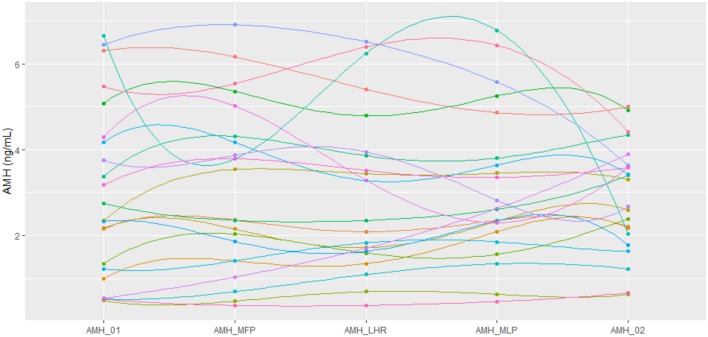
Representation of AMH values using a polynomial-exponential interpolation of data for each patient at every time point. AMH_01: day 2/3 of cycle. AMH_MFP: day 10 of cycle, mid follicular phase. AMH_LHR: day of LH rise. AMH_MLP: day 8 after LH rise, mid luteal phase. AMH_02: day 2/3 of the subsequent cycle.

The values of serum AMH concentrations obtained during the different phases of the menstrual cycle are shown in Table 2 and the fluctuations presented in AMH levels for each participant are represented in Figure 1. A significant longitudinal fluctuation in AMH levels was found per participant, expressed as coefficient of variation (CV) intra-cycle of 20.7%. This was calculated for all individual readings per cycle day (CV = mean 0.207 ± SD 0.143; [CI95: 0.148, 0.267]; Shapiro test = 0.0016). No pattern of fluctuation was observed for these variations.

**Table 2:** AMH values obtained each day of measurement for all the 22 participants, including the mean, minimum value and maximum value.

**ID_PARTICIPANT**	**AMH_01**	**AMH_MFP**	**AMH_LHR**	**AMH_MLP**	**AMH_02**	**MEAN, (MIN–MAX)**
ID001	6.3	6.16	5.41	4.86	-	5.68 (4.86–63.)
ID002	2.17	2.36	2.09	2.35	2.2	2.23 (2.09–2.36)
ID003	0.99	1.41	1.34	2.09	2.18	1.60 (0.99–2.18)
ID004	2.15	2.15	1.72	2.33	2.59	2.19 (1.72–2.59)
ID005	2.34	3.54	3.44	3.45	3.3	3.21 (2.34–3.54)
ID006	0.48	0.47	0.69	0.63	0.62	0.57 (0.47–0.62)
ID007	1.34	2.03	1.59	1.56	2.39	1.78 (1.34–2.39)
ID008	5.08	5.36	4.79	5.25	4.91	5.08 (4.79–5.36)
ID009	2.74	2.35	2.35	2.61	–	2.51 (2.35–2.74)
ID010	3.38	4.31	3.86	3.8	4.34	3.94 (3.38–4.34)
ID011	6.66	3.78	6.24	6.78	–	5.86 (3.78–6.78)
ID012	0.52	0.69	1.09	1.34	1.22	0.97 (0.52–1.34)
ID013	1.22	1.41	1.83	1.85	1.63	1.59 (1.22–1.85)
ID014	4.17	4.17	3.28	3.64	3.42	4.57 (3.28–4.17)
ID015	2.33	1.86	1.63	2.34	1.77	1.99 (1.63–2.34)
ID016	6.44	–	–	5.57	3.63	5.21 (3.63–6.44)
ID017	3.76	3.87	3.95	2.82	2.67	3.41 (2.67–3.95)
ID018	3.15	–	–	2.63	3.9	3.23 (2.63–3.9)
ID019	4.29	–	3.29	2.3	3.59	3.37 (2.3–4.29)
ID020	3.18	3.79	–	3.35	3.58	3.48 (3.18–3.79)
ID021	0.51	0.37	0.37	0.46	0.66	0.47 (0.37–0.66)
ID022	5.47	5.54	6.39	–	4.42	5.46 (4.42–6.39)
	**AMH_01**	**AMH_MFP**	**AMH_LHR**	**AMH_MLP**	**AMH_02**	**TOTAL**
N	22	19	19	21	19	100
AMH ng/mL (mean ± SD)	3.12 ± 2.01	2.93 ± 1.74	2.91 ±1.82	2.95 ± 1.61	2.79 ± 1.26
CI.95	[2.26, 3.98]	[2.14, 3.71]	[2.09, 3.73]	[2.26, 3.64]	[2.23, 3.36]

*N, number of samples analyzed each day-group and total number of samples analyzed; Missing data shown; Lower part of the table, AMH mean values each time-point, including standard deviation; CI, confidence interval 95%; AMH_01, day 2/3 of cycle; AMH_MFP, day 10 of cycle mid follicular phase; AMH_LHR, day of LH rise; AMH_MLP, day 8 after LH rise mid luteal phase; AMH_02, day 2/3 of the subsequent cycle*.

Comparing the results between day 2/3 of cycle in both consecutive cycles, mean absolute inter-cycle variation of AMH was 0.75 ng/mL, with a range from 0.03 to 2.81 ng/mL. Inter-cycle CV was 0.28 (CI95: 0.16-0.39; *p* < 0.0001). Hence, in our participants, a 28% of variation between the AMH values measured on day 2/3 of two consecutive menstruations was seen.

**Figure 2:**
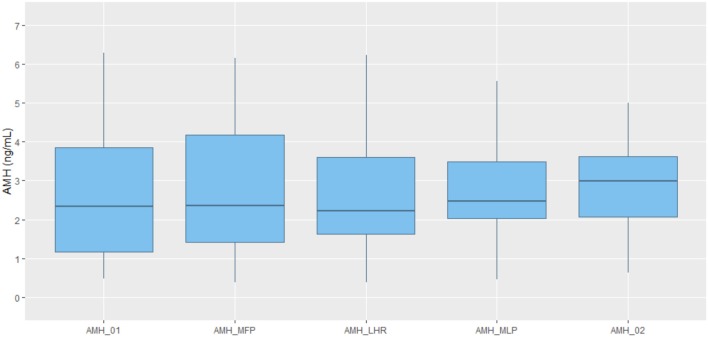
Boxplot representation of AMH values obtained at each time point. Line in the middle of the box representing AMH median value. Box representing 25 and 75% quartile. Upper whisker extends from the hinge to the largest value no further than 1.5^*^IQR (inter-quartile range). Lower whisker extends from the hinge to the lowest value. AMH_01: day 2/3 of cycle. AMH_MFP: day 10 of cycle, mid follicular phase. AMH_LHR: day of LH rise. AMH_MLP: day 8 after LH rise, mid luteal phase. AMH_02: day 2/3 of the subsequent cycle.

The average of the AMH values obtained on each day of measurement for all the 22 participants varies significantly through the cycle as shown by the data in Table 2, as well as represented in boxplot graph (Figure 2), where the intra-cycle variation and inter-cycle variation are shown.

### Other reproductive hormones related with amh variability.

All hormone values for AMH, FSH, LH, estradiol and progesterone are shown in Table 3. No correlation between AMH and FSH, estradiol and progesterone was observed at any time of the measurements. However, a significant positive correlation between LH_LHR and AMH concentrations was found (*t*-value: 5.962; *p* < 0.0001).

**Table 3:** AMH, FSH, LH, E2, and P4 values obtained each day of measurement for all the 22 participants.

**SUBJECT**	**AMH_01**	**P4_01**	**E2_01**	**LH_01**	**FSH_01**	**AMH_MPF**	**P4_MPF**	**E2_MPF**	**LH_MPF**	**FSH_MPF**	**AMH_LHR**	**P4_LHR**	**E2_LHR**	**LH_LHR**	**FSH_LHR**	**AMH_MLP**	**P4_MLP**	**E2_MLP**	**LH_MLP**	**FSH_MLP**	**AMH_02**	**P4_02**	**E2_02**	**LH_02**	**FSH_02**
**ID001**	**6.3**	0.45	40.48	3.46	8.4	**6.16**	0.38	86.11	9.9	7.15	**5.41**	0.61	241.6	84.99	15.9	**4.86**	17.14	129.9	7	5.07	**–**	**–**	**–**	**–**	**–**
**ID002**	**2.17**	0.74	33.4	2.72	7.64	**2.36**	0.63	59.03	7.41	6.8	**2.09**	1.14	92.82	17.36	8.35	**2.35**	10.6	101	2.5	3.19	**2.2**	0.6	32.25	2.63	6.95
**ID003**	**0.99**	0.46	83.75	4.81	4.15	**1.41**	1.47	59.76	8.13	6.67	**1.34**	2.81	84.74	12.78	5.52	**2.09**	7.99	65.57	1.88	2.34	**2.18**	0.6	59.82	4.22	4.96
**ID004**	**2.15**	0.21	33.68	4.34	9.56	**2.15**	0.2	126.1	10.85	6.4	**1.72**	2.6	57.85	15.33	3.72	**2.33**	10.13	96.01	4.03	3.22	**2.59**	0.4	26.48	3.37	6.2
**ID005**	**2.34**	0.54	28.16	3.44	8.01	**3.54**	0.53	90.97	7.08	5.84	**3.44**	0.86	258.3	59.93	22.44	**3.45**	20.92	163.2	1.91	2.3	**3.3**	0.53	26.41	2.43	7.16
**ID006**	**0.48**	0.52	20.86	4.89	10.9	**0.47**	0.63	58.71	7.38	5.25	**0.69**	0.57	155.2	16.92	5.25	**0.63**	11.38	60.63	7.3	5.94	**0.62**	0.55	24.21	4.56	7.59
**ID007**	**1.34**	0.36	33.77	3.26	7.77	**2.03**	0.45	54.37	5.55	6.28	**1.59**	0.59	229.2	44.31	15.64	**1.56**	12	160.7	1.5	3.08	**2.39**	0.39	31.24	3.4	6.76
**ID008**	**5.08**	0.6	47.04	6.23	5.09	**5.36**	0.66	123.9	6.86	3.94	**4.79**	0.98	314.2	50.96	9.89	**5.25**	18.59	239.9	2.63	2.15	**4.91**	0.48	44.01	9.73	4.97
**ID009**	**2.74**	0.53	48.79	5.31	8.81	**2.35**	0.73	306.8	21.95	12.25	**2.35**	0.81	186.2	37.73	11.12	**2.61**	16.56	347.4	1.55	2.22	**–**	**–**	**–**	**–**	**–**
**ID010**	**3.38**	0.39	33.89	4.66	7.17	**4.31**	0.4	313	7.46	4.35	**3.86**	0.77	394.8	45.22	13.5	**3.8**	15.74	227	4.53	2.51	**4.34**	0.37	35.16	2.75	7.01
**ID011**	**6.66**	0.27	75.87	7.29	9.41	**3.78**	0.24	310.3	7.61	5.79	**6.24**	0.76	515	52.9	17.22	**6.78**	6.35	175.9	2.04	2.52	**–**	**–**	**–**	**–**	**–**
**ID012**	**0.52**	0.2	43.5	7.9	12.44	**0.69**	0.19	184.6	8.25	5.95	**1.09**	0.92	214.2	13.42	6.5	**1.34**	5.88	66.52	3.16	3.59	**1.22**	0.05	36.68	9.19	11.17
**ID013**	**1.22**	0.05	29.31	4.68	10.55	**1.41**	0.05	28.41	10.68	9.31	**1.83**	0.17	151.2	31	12.15	**1.85**	9.11	106	8.18	3.96	**1.63**	0.05	28.29	3.86	8.36
**ID014**	**4.17**	0.6	47.71	4.81	6.19	**4.17**	0.18	261.4	11.18	5.91	**3.28**	1.32	143.4	15.81	7.34	**3.64**	13.55	97.15	3.9	3.86	**3.42**	0.43	53.56	3.75	6.78
**ID015**	**2.33**	0.05	28.52	6.03	5.68	**1.86**	0.05	295.8	10	3.16	**1.63**	0.44	409.9	25.16	5.1	**2.34**	22.78	179.7	6.42	1.95	**1.77**	0.21	17.72	7.31	7.34
**ID016**	**6.44**	0.14	63.15	6.79	8.27	**–**	**–**	**–**	**–**	**–**	**–**	**–**	**–**	**–**	**–**	**5.57**	14.1	82.39	2.63	3.39	**3.63**	0.15	25.78	2.88	8.21
**ID017**	**3.76**	0.11	19.28	4.94	5.15	**3.87**	50.86	0.05	8.66	4.25	**3.95**	0.16	226.6	21.19	4.39	**2.82**	8.43	112.9	2.02	2.16	**2.67**	0.31	23.35	8.41	6.24
**ID018**	**3.15**	0.2	45.5	3.15	6.34	**–**	**–**	**–**	**–**	**–**	**–**	**–**	**–**	**–**	**–**	**2.63**	13.54	107.9	1.86	3.26	**3.9**	0.13	37.28	3.8	5.84
**ID019**	**4.29**	0.19	13.89	7.03	5.41	**–**	**–**	**–**	**–**	**–**	**3.29**	0.08	78.55	25.57	5.77	**2.3**	6.9	66.16	11.22	3.5	**3.59**	0.29	24.31	9.25	5.84
**ID020**	**3.18**	0.06	19.79	6.14	7.17	**3.79**	0.05	70.93	11.31	7.11	**–**	**–**	**–**	**–**	**–**	**3.35**	10.59	118.1	4.59	3.52	**3.58**	0.16	24.53	5.31	6.56
**ID021**	**0.51**	0.06	5	4.39	16.99	**0.37**	0.14	275.4	9.3	13.82	**0.37**	0.34	386.4	29.13	14.2	**0.46**	16.07	107	5.26	4.21	**0.66**	0.14	5	5.11	14.4
**ID022**	**5.47**	0.05	19.28	7.78	5.01	**5.54**	0.05	157.86	13.45	6.02	**6.39**	1.55	131.3	29.52	6.38	**–**	**–**	**–**	**–**	**–**	**4.42**	0.05	22.07	7.29	5.96

*Missing data show (Units: AMH ng/mL, P4 ng/mL, E2 pg/mL, LH mIU/mL, FSH mIU/mL)*.

## Discussion

One of the advantages of AMH as compared to other measures of ovarian reserve such as follicle stimulating hormone (FSH) is that it is thought to have minimal variation within the menstrual cycle and as a result, it can be tested on any day of the cycle. However, studies assessing the stability of AMH levels throughout the menstrual cycle have been conflicting and difficult to interpret due to the differences in assays used. The current study clearly demonstrated a statistically significant variation in serum AMH levels using a fully automated AMH assay during a spontaneous menstrual cycle in a group of healthy reproductive aged women. This finding is in keeping with other previous studies (27–30), some of which identified a particular pattern of change with AMH levels at their highest in the mid-follicular phase, decreasing at the time of ovulation with a rise again in the luteal phase (16, 28, 29, 31). This pattern has been suggested to reflect the role of AMH in follicular growth (32). The current analysis revealed that women, regardless of their ovarian reserve, exhibited a statistically significant intra- and inter-cycle AMH variation, however, a clear fluctuation pattern could not be identified. Notably, a significant positive co-variation between serum levels of AMH and LH was observed during the LH rise. This association between LH and AMH levels was described as well by Bungum et al. (18), when they investigated circadian variations of serum AMH. This correlation between both hormones points to a joint regulatory mechanism between AMH and the secretion of LH, supported by the recent finding of AMH receptors in significant subset of GnRH central neurons in mice and humans (33), which should encourage further research.

For the women included, AMH values varied 20.7% throughout the cycle from the first serum analysis obtained at the beginning of the cycle to day 2/3 of the consecutive menses. These AMH fluctuations are more than three times higher than the expected values attributed to assay imprecision (<5% assay imprecision expected for Elecsys® AMH automated assay) and may, therefore, be reflecting biological fluctuations. Intra-individual variations have been also demonstrated using different assays for the serum analysis (34).

The evidence to date regarding variability of AMH across the menstrual cycle is conflicting (19). Some of these discrepancies may be explained by inclusion of anovulatory cycles, infrequent blood sampling, sample handling or assay interference. Related to that, the present study has several strengths. All blood samples per study subject were assayed in one run on the same day in the same Cobas Elecsys platform, thereby reducing analytical variability and confounding factors. Future studies may consider as well repeating the analysis of the samples on a separate Cobas Elecsys platform for a quality assurance validation. Another important strength is that an average of 4.5 samples per participant were analyzed throughout the natural cycle and ovulation was confirmed by measurement of serum LH and luteal phase progesterone concentrations in all cycles, avoiding the low accuracy of the urine tests to detect ovulation. In addition, a fully automated AMH assay was used for sample analysis in this study, which has previously shown to have low analytical variability across the assay range (22, 35). All the samples were obtained in the morning or early afternoon, to avoid possible circadian variations (18). However, our number of study participants is limited.

The intra-cycle variations in healthy women identified are similar to the data published by Hadlow et al., which revealed an average intra-individual variation of AMH concentration of 20% (combined analytical and biological) in infertile women with ovulatory cycles during a study period of 3 cycles (16). In contrast, the participants of the present study are a representative sample of reproductive aged women with ovulatory cycles. Furthermore, a significant intra-individual variation was identified in a shorter period of time (one ovulatory cycle). The extent of AMH-variation in the current analysis is higher than previously described by Lambert-Messerlian, who concluded that despite a variation of 16% throughout the cycle, sample collection could be performed on any day for assessment of ovarian reserve (15). The variability of more than 20% with a fully automated AMH assay in the present study ought to be a reminder to clinicians that sampling AMH on any day of the menstrual cycle may not adequately reflect the true ovarian reserve.

It is apparent that AMH levels fluctuate not only within cycles, but also between cycles. An inter-cycle variability of 28% (*p* < 0.0001) between 2 consecutive cycles was identified in the present study. Furthermore, 4 participants with AMH levels <1.2 ng/mL on day 2/3 of the first cycle (AMH_01), would have been categorized as having a low AMH based on the Bologna Criteria (36). Two of these four volunteers showed normal AMH levels after reassessing on day 2/3 of the consecutive cycle (AMH_02). These differences in consecutive cycles may be a reflection of variances in antral follicle counts (AFC). It may also reflect the different ovarian response that can be seen in clinical practice despite prescribing the same treatment protocol to the same patient but in a different cycle (37, 38). This is a very important finding, which could change current clinical practice in terms of AMH testing. Inter-cycle assessment of serum AMH on day 2/3 certainly merits further study.

While real ovarian reserve may not vary during a natural cycle or between consecutive cycles, serum AMH levels do, due to the biological variation and, in addition, unusual AMH isoforms that have been described as well (39). It is clear that AMH is not an easy molecule to quantify. However, important clinical decisions are often based on one random AMH recording, even the dose of gonadotropins to be prescribed in IVF/ICSI cycles (34).

Previous publications have shown that AMH measurement when performed during the first few days of the cycle correlates well with ovarian response to stimulation (basal AMH) (7, 8). Additionally, a combined evaluation of serum AMH and ultrasound evaluation of antral follicle count (AFC) during the first days of the menstrual cycle is recommended prior to counseling of women regarding their reproductive health and the ovarian stimulation outcomes (8, 40). By combining both ovarian markers, AMH and AFC on day 1-3 of cycle, clinicians will be able to determine with greater accuracy the developing follicles expected to grow further following hormone stimulation. This will enable the clinicians to prescribe the most appropriate dosage of gonadotropins for ovarian stimulation at any given cycle, offering even more individualized treatment.

In summary, this study clearly demonstrated significant intra- and inter-cycle variations in serum AMH concentrations throughout natural ovulatory cycles and between two consecutive menstruations using a fully automated AMH assay. Based on these results it is reasonable to question current clinical practice of using a single random AMH measurement in determining ovarian reserve.

## Author contributions

The authors listed on this manuscript substantially contributed to the study conception and design, acquisition and interpretation of data, and drafting/revising the manuscript. All have given final approval of the version to be published.

### Conflict of interest statement

Authors are nowadays employed by IVIRMA. LM, BL, JS, EA, CC, and HF in IVIRMA Middle East—UAE and AN in IVI Foundation—IVIRMA Valencia (Spain).
